# An Unusual Presentation of Spinal Tuberculosis Masquerading as Pyogenic Meningitis: A Case Report

**DOI:** 10.1155/carm/4815700

**Published:** 2026-02-08

**Authors:** Parackrama Karunathilake, Sanjeewa Bowatte, Prabhashini Kumarihamy, Dushantha Madegedara, Madushika Gunathilake, Beruni Narmada

**Affiliations:** ^1^ Department of Medicine, Faculty of Medicine, Wayamba University of Sri Lanka, Kuliyapitiya, Sri Lanka, wyb.ac.lk

**Keywords:** infective spondylitis, neutrophilic pleocytosis, paraplegia, pyogenic meningitis, spinal tuberculosis

## Abstract

**Background:**

Spinal tuberculosis (TB), though accounting for up to 50% of skeletal TB cases, typically presents insidiously with back pain, kyphotic deformity, or neurological deficits. Atypical presentations, including initial neutrophilic pleocytosis in cerebrospinal fluid (CSF), can mimic bacterial meningitis and complicate diagnosis.

**Case Presentation:**

We report the case of a 46‐year‐old previously healthy male who initially presented with high‐grade fever, headache, neck stiffness, and neutrophilic CSF pleocytosis suggestive of bacterial meningitis. Despite empirical antibiotic therapy, the patient developed progressive neurological symptoms, including paraplegia and hypertonia. MRI of the spine revealed T2–T6 vertebral body involvement with compressive myelopathy consistent with spinal TB. CSF PCR for *Mycobacterium tuberculosis* was positive. Anti‐TB therapy and adjunct corticosteroids were initiated, leading to marked clinical improvement.

**Conclusion:**

Clinicians should maintain a high index of suspicion for spinal TB in patients with persistent fever, neurological symptoms, and CSF pleocytosis—especially in TB‐endemic regions—even when initial laboratory findings suggest bacterial meningitis. Early imaging and targeted therapy can significantly improve outcomes.

## 1. Introduction

Tuberculosis (TB) is a chronic infectious disease mainly caused by *Mycobacterium tuberculosis* and, less commonly, by *Mycobacterium bovis* [1]. Evidence of spinal TB has been identified in skeletal remains from the Iron Age in Europe, as well as in mummies from ancient Egypt and South America, highlighting its long‐standing presence in human history [2]. Spinal TB usually develops as a secondary manifestation of infection most often originating from a pulmonary focus documented in nearly 50% of the affected patients [3]. Nearly two billion people are infected with TB worldwide. However, only 5%–15% of them develop symptoms, while the rest remain in a latent state [4]. Among extrapulmonary TB cases, around 10% involve the skeletal system, with the spine being the most affected site accounting for nearly 50% of all skeletal TB cases [5].

The infection usually reaches the spine through hematogenous dissemination from a primary focus elsewhere in the body [6]. The anterior vertebral bodies of the thoracic and lumbar spine are commonly involved in the disease process [3]. The clinical course is typically slow and progressive, with cold abscess formation, neurological impairment, and kyphotic deformity being the principal manifestations, especially in advanced or untreated disease [2]. Neurological deficits occur in approximately 10%–20% of spinal TB cases in developed countries, with prevalence being twice as high in developing nations [7].

Treatment strategies for spinal TB include both medical therapy and surgical intervention, aiming to eradicate infection, prevent drug resistance, preserve neurological function, correct or limit deformity, and reduce the likelihood of recurrence.

The treatment of spinal TB include both medical and surgical interventions, with the primary goals being curing the infection, preventing the development of drug‐resistant tuberculosis, minimizing disability caused by neurological deficits or kyphosis, and reducing the likelihood of recurrence. With current treatment approaches, favorable outcomes are achieved in 85%–95% of patients, even among those presenting with neurological deficits.

Spinal TB has a generally favorable outcome with current treatment modalities, with 85%–95% showing improved outcomes even when presented with neurological deficits [2, 8]. We report a case of spinal TB presenting with paraplegia, confirmed radiologically, which demonstrated marked neurological improvement following anti‐TB therapy.

## 2. Case Presentation

A 46‐year‐old previously healthy male electrical technician presented initially to a general practitioner with an insidious onset of high‐grade continuous fever, associated with chills and rigors, which was unresponsive to paracetamol. He also reported a productive cough, significant headache, and arthralgia. Notably, he denied abdominal pain, urinary symptoms, cellulitis, seizures, or loss of appetite. After 1 week of unsuccessful outpatient treatment, he sought further care at a local hospital while having low grade fever and headache.

Upon admission to the hospital, he was clinically stable with a blood pressure of 100/60 mmHg and a pulse rate of 96 beats per minute and was conscious with a Glasgow Coma Scale (GCS) of 15/15. He had neck stiffness with positive Kernig’s and Brudzinski’s sign; however, other neurological findings were normal. He had bilateral rhonchi on respiratory examination, and the cardiovascular and abdominal examination findings were unremarkable.

The laboratory investigations revealed a white cell count of 22.54 × 10^9^/L with neutrophil predominance (18.62 × 10^9^/L). The inflammatory markers were elevated with a C‐reactive protein (CRP) level of 32.87 mg/L and an erythrocyte sedimentation rate (ESR) of 124 mm/h. The serum creatinine level was 0.84 mg/dL. A lumbar puncture was performed suspecting the diagnosis of meningitis where the cerebrospinal fluid (CSF) analysis indicated a predominantly neutrophilic response (granulocytes 250 cells/mm^3^ and lymphocytes 12 cells/mm^3^), raised protein (132 mg/dL), and a glucose level of 54 mg/dL while having a random blood sugar of 130 mg/dL. Serum electrolyte testing indicated hypernatremia, with sodium levels peaking at 153 mmol/L. The other blood investigations revealed an aspartate transaminase level of 19.9 U/L, alanine transaminase 15.5 U/L, alkaline phosphatase 66.2 U/L, and gamma glutamyl transferase 20.3 U/L. The albumin level was 3.8 g/dL. Total bilirubin was measured at 0.79 mg/dL, with direct bilirubin at 0.34 mg/dL and indirect bilirubin at 0.46 mg/dL. Microbiological evaluations, including blood, urine, and CSF cultures, yielded no microbial growth. Imaging at this stage, including noncontrast and contrast‐enhanced CT scans of the brain, was unremarkable. Although the markedly elevated ESR was more suggestive of tuberculosis, the initial clinical picture strongly supported an acute pyogenic process because the patient had insidious onset of high‐grade fever with chills and rigors, severe headache, neck stiffness with positive meningeal signs, and a neutrophil‐predominant CSF pleocytosis with elevated protein. These features, together with the absence of classical constitutional symptoms of TB such as weight loss, anorexia, or chronic cough, led to bacterial meningitis being considered the most likely initial diagnosis.

He was treated empirically with intravenous antibiotics (ceftriaxone, vancomycin, and meropenem). Following clinical improvement, the patient was discharged although he has developed some neurological impairments while on the hospital stay including limb weakness, hypertonia, and exaggerated tendon reflexes. These neurological findings were attributed to residual effects of meningoencephalitis and he was advised to continue outpatient physiotherapy and regular follow‐ups in medicine and cardiology clinics.

Two months later, due to minimal improvement and persistent neurological symptoms, the patient was readmitted to a tertiary care center with recurrence of fever lasting 2 weeks, generalized body aches, intermittent altered consciousness, involuntary movements of the bilateral upper limbs, severe imbalance, and difficulty ambulating. Clinical examination revealed marked hypertonia in all limbs, myoclonic jerks in bilateral upper limbs, exaggerated deep tendon reflexes in all four limbs, and positive jaw jerk, indicative of upper motor neuron involvement. All other cranial nerve examinations were unremarkable.

**Figure 1:**
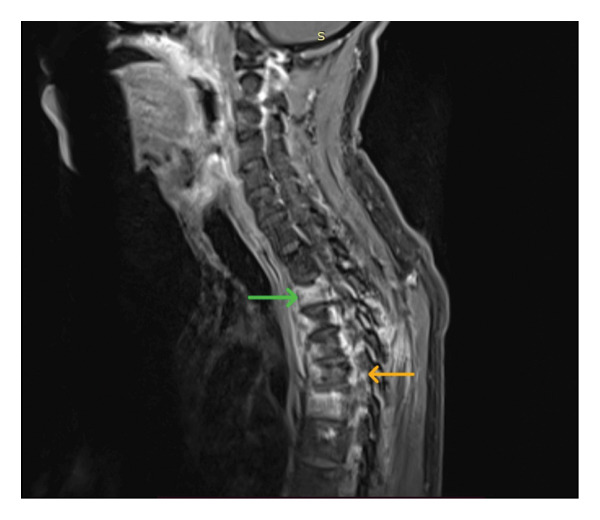
Sagittal T2‐weighted MRI of the spine demonstrating features consistent with infective spondylitis. The green arrow at the T2 vertebral body indicates marrow edema (T2 hyperintensity), while the orange arrow at the T5 level demonstrates spinal canal narrowing with resultant spinal cord compression and associated myelopathic signal change.

Repeat investigations during this second admission the investigations revealed a white cell count of 7.45 × 10^9^/L, with neutrophils 60.0% and lymphocytes 25.1%. The ESR was 94 mm/h with a CRP of 7.0 mg/dL. A repeat CSF analysis was performed with clear CSF, 7 lymphocytes per mm^3^, and no polymorphs and a protein level of 134 mg/dL. An electroencephalogram (EEG) was performed which was normal. Given the clinical picture and persistent symptoms, an MRI of the spine was performed which demonstrated features of infective spondylitis involving the thoracic vertebrae with vertebral body marrow edema, spinal canal narrowing at the T5 level causing spinal cord compression, and associated myelopathic signal changes (Figure 1). Degenerative cervical spine changes with additional myelopathic features were also noted. MRI brain, as reported by the consultant radiologist, demonstrated basal meningeal enhancement suggestive of chronic inflammatory changes. However, the MRI brain images were not available for inclusion in this report.

**Figure 2:**
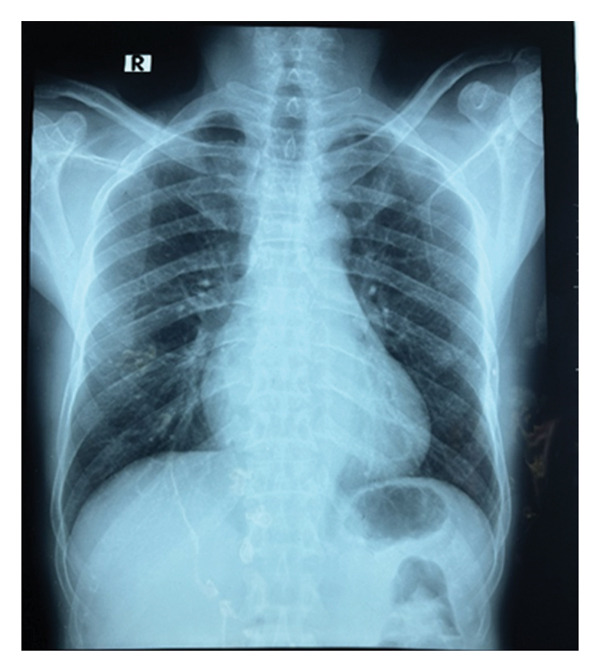
Chest x‐ray was unremarkable.

Given the chronicity and atypical presentation, a detailed evaluation for possible etiologies was conducted. The patient had no known history of tuberculosis contact, and there was no family or occupational exposure to TB. The chest radiograph was unremarkable (Figure 2). Brucellosis was considered due to the prolonged fever and neurologic symptoms, but there was no history of animal contact or occupational exposure, making the diagnosis less likely. Sarcoidosis was excluded on the basis of normal serum calcium levels and absence of supportive imaging findings. A lymphoproliferative disorder such as lymphoma was considered; however, the peripheral blood smear and full blood count were unremarkable, and there was no lymphadenopathy. Autoimmune etiologies, including connective tissue diseases, were excluded based on lack of clinical features such as rash, arthritis, or serositis and absence of positive serology. There were no clinical signs or laboratory evidence suggestive of systemic vasculitis. Finally, HIV infection was ruled out with negative serological testing. These exclusions, along with the MRI and CSF findings, guided the revised diagnosis toward spinal tuberculosis with secondary meningoencephalitis. Subsequently the tuberculosis PCR of the CSF was positive. The patient promptly initiated anti‐TB therapy with a fixed‐dose, WHO‐recommended first‐line regimen (Category I: HRZE), adjunct corticosteroids (prednisolone 30 mg daily), vitamin B6 supplementation, alendronate, and calcium lactate and was referred to neurosurgery and orthopedics for further management, including decompressive surgery and spinal stabilization. Surgical decompression was deferred at that stage, with the plan to re‐evaluate as needed, under close monitoring. The patient demonstrated clinical improvement following targeted therapy and multidisciplinary care. He was subsequently discharged with the plan to continue anti‐TB treatments for 12 months.

A timeline summarizing the chronological sequence of symptoms, investigations, and management of this patient is shown in Table 1.

**Table 1:** Timeline of clinical events, investigations, and treatments.

Day 0: symptom onset	• The patient, a 46‐year‐old previously healthy male, developed high‐grade continuous fever, headache, and neck stiffness. • No constitutional symptoms (weight loss, cough, or anorexia) were present.

Day 7: first admission	• Admitted with signs of meningitis. • Lumbar puncture: neutrophilic pleocytosis (granulocytes 250/mm^3^), elevated protein (132 mg/dL), normal glucose. • Investigations: ESR 124 mm/h, CRP 32.9 mg/L, CSF/blood cultures negative. • Imaging: brain CT normal. • Provisional diagnosis: bacterial meningitis ⟶ Empirical IV antibiotics (ceftriaxone, vancomycin, meropenem).

Day 14–60: progressive neurological deterioration	• Worsening weakness, hyperreflexia, imbalance, and myoclonic jerks despite physiotherapy. • No recurrence of meningitic symptoms but increasing spasticity.

Month 2: second admission	• MRI spine: T2–T6 vertebral body involvement, compressive myelopathy, early syrinx formation. • CSF: lymphocytic shift, protein 134 mg/dL, CSF PCR positive for Mycobacterium tuberculosis. • Other causes (brucellosis, sarcoidosis, lymphoma, autoimmune disease, HIV) were excluded. • Antituberculosis therapy (HRZE) + prednisolone 30 mg/day, vitamin B6, alendronate, calcium. • Gradual clinical improvement observed over the following weeks.

## 3. Discussion

Despite global TB control efforts, extrapulmonary manifestations remain common, with spinal involvement accounting for the majority of skeletal TB cases and posing a significant risk of neurological morbidity [2, 4].

Spinal TB typically results from hematogenous spread, often affecting the anterior vertebral bodies of the thoracic and lumbar spine [3, 6]. The disease progresses insidiously and can lead to serious complications, including vertebral collapse, paraspinal abscess formation, and neurologic deficits such as paraplegia if left undiagnosed or untreated [3, 9]. Constitutional symptoms such as low‐grade fever, malaise, weight loss, and anorexia are present only in a small proportion of spinal TB patients (20%–38%) [10]. In our case, the patient lacked classical constitutional symptoms, underscoring the importance of maintaining a high index of suspicion for TB in patients presenting with chronic back pain, especially in endemic areas.

Back pain is the most common presenting symptom of spinal TB, occurring in nearly all patients and sometimes as the sole symptom [11]. This pain may be localized or radicular depending on vertebral and nerve root involvement. Our patient presented with significant axial back pain but no systemic symptoms, further demonstrating how spinal TB can mimic benign or degenerative spinal conditions.

Neurological involvement in spinal TB varies widely, occurring in 10%–20% of the patients in developed countries and up to 76% in developing nations [6]. Thoracic involvement is associated with a higher risk of neurological complications due to the narrower spinal canal in this region [8]. Neurological deficits may range from subtle motor symptoms to complete paraplegia. Pathophysiological mechanisms include direct spinal cord compression by abscesses, granulomatous tissue, vertebral collapse, and inflammatory edema [5]. In our case, the onset of paraparesis and sphincter dysfunction indicated early cord compromise, necessitating urgent evaluation and intervention.

Residual neurological impairment is relatively uncommon in adequately treated bacterial meningitis, particularly when imaging and CSF findings are normal. Therefore, the persistence and progression of neurological signs which cannot be explained by meningeal involvement itself in our patient should have prompted earlier consideration of spinal TB as a differential diagnosis even in the absence of classic systemic symptoms [12].

Routine laboratory parameters lack diagnostic specificity in spinal TB. While leukocytosis may suggest infection, it is observed in only 30%–50% of spinal TB cases. Notably, TB is often associated with disproportionately elevated ESR (commonly > 100 mm/h), while CRP levels may be only mildly elevated. This contrasts with bacterial infections, in which both markers typically rise in tandem [5]. Our patient’s lab results mirrored this pattern.

The Mantoux test or tuberculin skin test (TST) may support the diagnosis, though they cannot distinguish between latent and active disease. TST has a positivity rate ranging from 63% to 90% in spinal TB [5]. Adenosine deaminase (ADA) testing in CSF is a valuable diagnostic tool for tuberculous meningitis, particularly in resource‐constrained settings. Although ADA was not performed in our patient, it has been shown to offer moderate sensitivity and specificity when interpreted alongside clinical and radiological features. ADA measurement should be considered in all suspected CNS TB cases, especially in endemic regions [13].

At the time of the first admission, neither CSF ADA nor GeneXpert MTB/RIF testing was performed in our patient due to the strong initial impression of acute bacterial meningitis and the limited availability of these assays in peripheral hospitals. In retrospect, performing ADA or GeneXpert at this stage may have provided an earlier clue toward TB, thereby potentially altering the diagnostic pathway and avoiding a delay in initiating targeted therapy.

While TB meningitis is classically associated with lymphocytic CSF pleocytosis, up to 25% of the cases may initially show neutrophilic predominance, particularly early in the disease course [14]. This was seen in our patient and led to a presumptive diagnosis of bacterial meningitis. Unfortunately, the initial neutrophilic response and lack of typical systemic features delayed the correct diagnosis, underscoring the diagnostic challenge in such presentations.

A few earlier reports similarly document cases in which tuberculous meningitis presented with neutrophil‐predominant CSF (“neutrophilic meningitis”), thereby masquerading as bacterial infections. Pinto et al. described three patients with persistent neutrophilic CSF whose final diagnoses were tuberculous meningitis [15]. Sternberg et al. reported concomitant spinal and brain tuberculosis in a patient presenting with meningitic features [16]. In another recent report, an individual developed acute paraplegia in the setting of tuberculous meningitis before the diagnosis was established [17]. Our case differs from these in combining early neutrophilic CSF, progression to paraplegia, and imaging evidence of vertebral involvement preceding classic systemic signs. This juxtaposition underscores the rarity and diagnostic difficulty of TB cases that blur the conventional distinctions between meningitis and spinal infection.

Imaging is essential for confirming spinal TB. Although radiographs may show lytic vertebral lesions and reduced disc height, they lack sensitivity in early disease [5]. MRI is the preferred imaging modality for suspected spinal TB because of its ability to delineate vertebral, paraspinal, and neural involvement with high diagnostic accuracy [5, 8]. In our case, differentiation from pyogenic spondylitis and neoplastic lesions was important. Pyogenic spondylitis usually demonstrates involvement of a single vertebral body with early disc destruction and prominent paravertebral abscesses, whereas TB often involves multiple contiguous vertebrae with relative sparing of intervertebral discs until later stages [18]. Neoplastic lesions, in contrast, tend to show vertebral body collapse without significant paraspinal soft tissue involvement and typically lack the classical epidural or paravertebral abscesses seen in TB [19]. The MRI findings in our patient (contiguous involvement of T2–T6 vertebral bodies, preserved disc spaces, paraspinal inflammatory changes, and cord compression with early syrinx formation) were characteristic of spinal TB and not consistent with pyogenic or neoplastic etiologies.

Given the paucibacillary nature of spinal TB, microbiological confirmation through CSF or tissue culture is often limited, particularly after empirical antibiotic use [8]. Although imaging‐guided biopsy and TB culture were possible diagnostic options, we did not pursue these investigations given the confirmed diagnosis through other supportive investigations and the limited availability of resources.

Treatment is primarily medical. Current treatment recommendations advocate a 6‐month anti‐tubercular regimen comprising an initial intensive phase with four first‐line agents followed by a continuation phase with two drugs, aiming to achieve bacteriological cure and prevent neurological deterioration [20]. Early initiation of treatment is crucial to preventing progression, neurologic compromise, and deformity. Surgical intervention is typically reserved for cases with severe neurologic deficits, spinal instability, or failure of medical therapy. Surgical goals include decompression, abscess drainage, spinal stabilization, and deformity correction [7].

Although the MRI demonstrated spinal cord compression at T5 and early syrinx formation, surgical decompression was not undertaken immediately in our patient for several reasons. First, at the time of diagnosis, the patient’s neurological deficits had not progressed to complete paraplegia, and the multidisciplinary team judged that conservative medical therapy with antitubercular treatment and steroids might lead to regression of inflammatory edema and reduce compressive lesion burden. Second, given the patient’s clinical stability and the risks associated with thoracic spinal surgery (particularly in a resource‐constrained setting), we opted for a cautious “watchful decompression” strategy with close neurological monitoring. Previous case series have shown that in selected patients with early neurological signs, nonoperative management can succeed, with resolution of epidural inflammation and avoidance of surgery, provided deterioration is closely watched [21].

## 4. Conclusion

This case highlights the diagnostic challenges posed by spinal tuberculosis, particularly in its atypical presentations. The absence of constitutional symptoms and an initial neutrophil‐predominant CSF profile mimicking pyogenic meningitis contributed to a delay in diagnosis. MRI played a pivotal role in identifying thoracic spinal involvement and cord compression, leading to the correct diagnosis. This underscores the importance of considering TB in the differential diagnosis of persistent or atypical CNS infections, especially in endemic areas when the patient has unexplained solid clinical signs. Early recognition and prompt initiation of anti‐TB therapy remain essential to prevent irreversible neurological damage and deformity.

**Nomenclature**
TB: Tuberculosis
CSF: Cerebrospinal fluid
MRI: Magnetic resonance imaging
PCR: Polymerase chain reaction
CRP: C‐reactive protein
ESR: Erythrocyte sedimentation rate
GCS: Glasgow Coma Scale
CT: Computed tomography
EEG: Electroencephalogram
MTB: Mycobacterium tuberculosis
ADA: Adenosine deaminase
TST: Tuberculin skin test
WHO: World Health Organization
HRZE: Isoniazid, rifampicin, pyrazinamide, ethambutol

## Author Contributions

All authors were involved in the management of the patient and generating the concept. All authors made an intellectual contribution and wrote the paper.

## Funding

This study was self‐funded by the investigators. No external organization or institution was involved in this study.

## Disclosure

All authors read and approved the final manuscript.

## Ethics Statement

The authors have nothing to report.

## Consent

Informed written consent was obtained from the relatives of the patient for publication of this case report.

## Conflicts of Interest

The authors declare no conflicts of interest.

## Data Availability

The authors confirm that the data supporting the findings of this study are available within the article.
